# Determinants and predictive performance of reduced muscle mass in elderly patients with type 2 diabetes: a retrospective study

**DOI:** 10.3389/fendo.2026.1746797

**Published:** 2026-03-04

**Authors:** Kaili Wang, Weitao Fu, Haiyan Pan, Jianjun Dong

**Affiliations:** 1 The First Clinical Medical College, Cheeloo College of Medicine, Shandong University, Jinan, Shandong, China; 2 Department of Neurosurgery, Qilu Hospital, Cheeloo College of Medicine and Institute of Brain and Brain-Inspired Science, Shandong University, Jinan, Shandong, China; 3 Department of Endocrinology and Metabolism, Qilu Hospital of Shandong University, Jinan, Shandong, China

**Keywords:** discriminatory power, risk factors, sarcopenia, skeletal muscle mass, type 2 diabetes mellitus

## Abstract

**Objective:**

This study aimed to identify risk factors for low muscle mass among elderly patients with type 2 diabetes mellitus (T2DM).

**Methods:**

In this cross-sectional study, 521 elderly T2DM patients were enrolled, comprising 253 with low muscle mass and 268 with normal muscle mass. Clinical characteristics were compared between groups and stratified by gender. Binary logistic regression was conducted to identify risk factors for muscle mass reduction. Receiver operating characteristic (ROC) curve analysis was performed to evaluate the predictive performance of relevant factors for low muscle mass.

**Results:**

Patients with reduced muscle mass were older and had lower body mass index (BMI) and waist-to-hip ratio (WHR). Age, BMI, and diabetic sensorimotor polyneuropathy (DSPN) were independently associated with low muscle mass in elderly T2DM patients across both genders. The odds ratios (*P* < 0.05) were 1.098, 0.590, and 2.334 for males, and 1.063, 0.681, and 3.621 for females, respectively. We also found insulin use was independently associated with a lower risk of low muscle mass in men, whereas sulfonylurea use was associated with a higher risk in women. Among the significant variables, BMI demonstrated the greatest discriminatory ability for identifying reduced muscle mass, with AUCs of 0.815(95%CI:0.763-0.859) in men and 0.763(95%CI:0.705-0.814) in women.

**Conclusions:**

Older age, lower BMI, DSPN, and the use of insulin and sulfonylureas were independently associated with reduced muscle mass in elderly patients with T2DM. BMI demonstrated the strongest discriminative capacity among all significant variables.

## 1 Introduction

China is facing dual demographic challenges characterized by a rapidly growing elderly population and an accelerating aging process (1), which together have led to an increased demand for a better quality of life. As life expectancy increases, the prevalence of age-related diseases, such as type 2 diabetes mellitus (T2DM), continues to rise. Although T2DM is now being diagnosed at younger ages, the elderly remain the most significantly affected group (2). Sarcopenia, a multifactorial condition strongly associated with aging, is characterized by low muscle mass, decreased muscle strength, and impaired physical function (3). According to the 2019 consensus of the Asian Working Group for Sarcopenia (AWGS), reduced muscle mass represents a core diagnostic criterion for sarcopenia (3). Sarcopenia often coexists with metabolic disorders, such as T2DM and cardiovascular diseases (4), significantly increasing the risk of adverse outcomes in older adults, including falls, fractures, and higher rates of hospitalization (5).

Older adults with T2DM are particularly vulnerable to sarcopenia due to multiple factors, including insulin resistance, physical inactivity, obesity, and restrictive dietary practices (6–8). These factors contribute to an increased risk of muscle mass reduction, thereby exacerbating the overall burden of both diabetes and sarcopenia. Despite increasing recognition of sarcopenia and substantial research on its pathogenesis, effective strategies for its prevention and intervention remain limited. The subtle and non-specific clinical manifestations of sarcopenia often lead to underdiagnosis in clinical practice. Moreover, routine assessments for sarcopenia, including muscle strength testing and physical performance evaluations, are seldom performed (9).

Given these challenges, the early identification of elderly patients with T2DM and low muscle mass is essential for implementing targeted interventions that can not only prevent sarcopenia but also improve glycemic control and overall clinical outcomes. However, previous studies have paid limited attention to the roles of sex, glucose-lowering medications, and bone metabolism–related markers in the development of sarcopenia (10–12). Moreover, the predictive performance of these risk factors has rarely been systematically evaluated. Thus, this study aims to address existing gaps and further identify the risk factors associated with low muscle mass in elderly patients with T2DM, particularly across sexes. The findings are expected to provide new clinical insights for the screening and prevention of sarcopenia in this high-risk population.

## 2 Materials and methods

### 2.1 Study design and participants

This retrospective cross-sectional study was conducted at Qilu Hospital of Shandong University (Jinan, China) between March and December 2024. The inclusion criteria were as follows: (1) age ≥ 60 years; and (2) a diagnosis of T2DM based on the 1999 World Health Organization (WHO) criteria (13). Exclusion criteria included: (1) age < 60 years; (2) diagnosis of type 1 diabetes mellitus (T1DM), gestational diabetes, or other specific types of diabetes; (3) presence of severe cardiovascular, hepatic, or renal disease, defined as an estimated glomerular filtration rate (eGFR) < 30 mL/min/1.73 m^2^; (4) history of diabetic ketoacidosis, hyperosmolar hyperglycemic state, or other acute diabetic complications; (5) severe malnutrition, including malignancy or hypoalbuminemia; and (6) disability or severe cognitive impairment that could interfere with study participation. In total, 521 elderly patients with T2DM were included in the final analysis.

### 2.2 Assessment of clinical characteristics

Data on demographic characteristics, medical history, and lifestyle factors were obtained from the participants’ electronic medical records. These variables included age, sex, duration of diabetes, smoking status, and alcohol consumption. Smoking was defined as daily or near-daily tobacco use, and alcohol consumption was defined as weekly or near-weekly drinking. Medical histories of hypertension and coronary artery disease (CAD) were also recorded. Hypertension was defined as systolic blood pressure (SBP) ≥140 mmHg or diastolic blood pressure (DBP) ≥90 mmHg (14).

#### 2.2.1 Body composition parameters

Body mass index (BMI) was calculated as weight (kg) divided by height squared (m^2^). Body fat percentage (BF%), appendicular skeletal muscle mass index (ASMI), waist-to-hip ratio (WHR), and bone mineral density (BMD) were measured using dual-energy X-ray absorptiometry (DXA; Hologic Discovery™ device, Waltham, MA, USA). BF% was calculated as total fat mass divided by total body mass × 100%. Low muscle mass was defined as an appendicular skeletal muscle mass (ASM)-to-height squared ratio <7.0 kg/m² in men and <5.4 kg/m² in women, according to the diagnostic criteria for sarcopenia(3). ASM was defined as the sum of lean mass of the four limbs measured by DXA. WHR was calculated as waist circumference divided by hip circumference. BMD was measured at two anatomical sites: the lumbar spine (L1-L4) and the left hip. Osteoporosis was defined according to the World Health Organization (WHO) criteria as a T-score ≤ -2.5 at either measurement site. When BMD results differed between anatomical sites, the diagnosis was based on the lowest T-score obtained, in accordance with standard clinical practice.

#### 2.2.2 Laboratory measurements

After an overnight fast of at least 8 hours, venous blood samples were collected by trained nurses. Glucose metabolism was evaluated using fasting plasma glucose (FPG), glycated hemoglobin (HbA1c), and fasting C-peptide (FCP). Lipid profiles were assessed by measuring total cholesterol (TC), triglycerides (TG), high-density lipoprotein cholesterol (HDL-C), and low-density lipoprotein cholesterol (LDL-C). Markers of bone metabolism, including serum calcium, intact parathyroid hormone (iPTH), osteocalcin (OC), β-C-terminal telopeptide of type I collagen (β-CTX), and N-terminal propeptide of type I procollagen (P1NP), were measured.

#### 2.2.3 Diabetic complications

The presence of diabetic complications, including diabetic sensorimotor polyneuropathy (DSPN), diabetic retinopathy (DR), and diabetic kidney disease (DKD), was also recorded. DSPN was diagnosed according to the following criteria: (1) a confirmed diagnosis of diabetes mellitus; (2) onset of neuropathy concurrent with or subsequent to the diagnosis of diabetes; (3) clinical manifestations consistent with typical DSPN features; and (4) exclusion of neuropathies attributable to other causes, including spinal disorders, cerebrovascular disease, or drug-induced neurotoxicity, particularly chemotherapy-related neuropathy (15). Renal function markers, including serum creatinine (SCr), blood urea nitrogen (BUN), and estimated glomerular filtration rate (eGFR), were evaluated. Serum albumin was measured as an indicator of nutritional status.

### 2.3 Statistical analysis

All statistical analyses were performed using SPSS software, version 29.0 (IBM Corp., Armonk, NY, USA). Continuous variables were expressed as mean ± standard deviation (SD), while categorical variables were presented as frequencies and percentages. Group differences in anthropometric and clinical parameters were examined using Student’s *t*-test for continuous variables and the chi-square test for categorical variables. Univariable logistic regression analysis was conducted to identify potential risk factors associated with low muscle mass. Variables with *P* < 0.10 in univariable analyses were entered into a multivariable binary logistic regression model to identify factors independently associated with reduced muscle mass. The discriminative performance of each independent factor was evaluated using receiver operating characteristic (ROC) curve analysis, with the area under the curve (AUC) calculated using MedCalc software (version 20.1; MedCalc Software Ltd., Ostend, Belgium). A two-tailed *P* value < 0.05 was considered statistically significant.

## 3 Results

A total of 521 elderly patients with T2DM were enrolled in this study, consisting of 267 men and 254 women. Among them, 253 (48.56%) were classified as having low skeletal muscle mass. The prevalence of low muscle mass was significantly higher in men (157 [62.06%]) than in women (96 [37.94%]; *P* < 0.001).

### 3.1 Clinical characteristics of participants with and without low muscle mass

#### 3.1.1 Overall baseline characteristics

Compared with patients without low muscle mass, those with low muscle mass were significantly older and had a higher prevalence of smoking. They also exhibited lower BMI, BF%, and WHR. With respect to metabolic and nutritional parameters, triglycerides and serum albumin levels were lower, whereas eGFR was higher in the low muscle mass group. In addition, patients with low muscle mass showed reduced levels of serum calcium, FCP, iPTH, and osteocalcin, accompanied by higher HbA1c levels. The prevalence of diabetic neuropathy was significantly greater in patients with low muscle mass. No significant differences were observed in alcohol consumption, BUN, β-CTX, P1NP, or thyroid function-related parameters. Regarding antidiabetic treatment, the use of insulin and dipeptidyl peptidase-4 (DPP-4) inhibitors was less frequent, whereas sulfonylurea use was more common in the low muscle mass group. Detailed results are presented in **Table 1**, **Supplementary Table 1**.

**Table 1:** Baseline characteristics of participants with and without low muscle mass.

Variables	Normal muscle mass	Low muscle mass	*P* value
N	268	253	
Male (n, %)	110(41.04%)	157(62.06%)	<0.001***
Age (years)	67.76 ± 5.56	69.56 ± 6.33	<0.001***
Duration of diabetes (years)	15.88 ± 7.76	16.96 ± 8.54	0.128
Smoking habit (n, %)	47(17.54%)	77(30.43%)	<0.001***
Drinking habit (n, %)	49(18.28%)	51(20.16%)	0.587
BMI (kg/m^2^)	26.42 ± 3.24	23.36 ± 2.54	<0.001***
BF%	32.55 ± 6.34	30.43 ± 6.50	<0.001***
WHR	1.15 ± 0.19	1.10 ± 0.18	0.005**
FPG (mmol/L)	7.53 ± 2.53	7.27 ± 2.76	0.287
TC (mmol/L)	4.26 ± 1.15	4.07 ± 1.12	0.056
TG (mmol/L)	1.70 ± 1.21	1.40 ± 1.11	0.004**
HDL-C (mmol/L)	1.14 ± 0.29	1.18 ± 0.32	0.222
LDL-C (mmol/L)	2.48 ± 0.88	2.38 ± 0.86	0.176
Serum albumin (g/L)	42.41 ± 3.31	41.33 ± 3.86	<0.001***
eGFR (mL/min/1.73m^2^)	86.09 ± 17.85	90.47 ± 18.39	0.006**
Serum creatinine (µmol/L)	70.94 ± 20.05	69.47 ± 22.11	0.424
Serum BUN (mmol/L)	6.29 ± 1.94	6.30 ± 2.09	0.954
HbA1c	8.49 ± 1.69	8.85 ± 1.94	0.022*
FCP (ng/mL)	1.75 ± 1.51	1.43 ± 0.97	0.005**
FT3 (pmol/L)	4.61 ± 0.59	4.59 ± 0.65	0.640
FT4 (pmol/L)	16.36 ± 2.80	16.54 ± 2.71	0.459
TSH (μIU/mL)	2.56 ± 5.15	1.97 ± 1.50	0.084
iPTH (pg/mL)	38.06 ± 14.84	34.39 ± 13.29	0.003**
Vitamin D (ng/mL)	19.49 ± 7.57	20.11 ± 8.36	0.375
Serum calcium (mmol/L)	2.30 ± 0.09	2.26 ± 0.10	<0.001***
Osteocalcin (OC) (ng/mL)	13.45 ± 5.58	12.43 ± 5.63	0.038*
DSPN (n, %)	132(49.25%)	185(73.12%)	<0.001***
DR (n, %)	162(60.45%)	143(56.52%)	0.363
DKD (n, %)	25(9.33%)	18(7.11%)	0.359
Osteoporosis (n, %)	55(20.52%)	63(23.51%)	0.233
Antidiabetic medication use			
Insulin (n, %)	167(62.31%)	100(39.53%)	<0.001***
Sulfonylureas (n, %)	77(28.73%)	110(43.48%)	<0.001***
DPP-4 inhibitors (n, %)	92(34.33%)	56(22.13%)	0.002**

CAD, coronary artery disease; BMI, body mass index; BF%, body fat percentage; WHR, waist-to-hip ratio; FPG, fasting plasma glucose; TC, total cholesterol; TG, triglycerides; HDL-C, high-density lipoprotein cholesterol; LDL-C, low-density lipoprotein cholesterol; eGFR, estimated glomerular filtration rate; BUN, blood urea nitrogen; HbA1c, glycated hemoglobin; FCP, fasting C-peptide; FT3, free triiodothyronine; FT4, free thyroxine; TSH, thyroid-stimulating hormone; iPTH, intact parathyroid hormone; DSPN, diabetic sensorimotor polyneuropathy; DR, diabetic retinopathy; DKD, diabetic kidney disease; DPP-4, dipeptidyl peptidase-4.

*represents *P* < 0.05, **represents *P* < 0.01, ***represents *P* < 0.001.

#### 3.1.2 Sex-specific baseline characteristics

After sex stratification, several baseline characteristics associated with low muscle mass in the overall analysis remained consistent in both men and women, including older age; lower BMI, WHR, and triglycerides; a higher prevalence of diabetic neuropathy; less frequent use of insulin and DPP-4 inhibitors; and more frequent use of sulfonylureas. Beyond these shared features, sex-specific analyses identified additional differences. Male patients with low muscle mass showed lower serum creatinine, FCP, and iPTH levels, whereas female patients had lower serum albumin and calcium levels, along with a higher prevalence of osteoporosis. The detailed data are shown in **Table 2**, **Supplementary Table 2**.

**Table 2:** Baseline characteristics of participants stratified by sex and skeletal muscle mass.

Variables	Male (n=267)			Female (n=254)		
	Normal muscle mass	Low muscle Mass	*P* value	Normal muscle mass	Low muscle mass	*P* value
N	110	157	--	158	96	--
Age (years)	65.86 ± 4.74	68.85 ± 6.17	<0.001***	69.08 ± 5.71	70.72 ± 6.44	0.035*
Duration of diabetes (years)	16.19 ± 7.68	16.49 ± 8.10	0.762	15.66 ± 7.83	17.75 ± 9.21	0.066
BMI (kg/m^2^)	26.71 ± 2.69	23.58 ± 2.37	<0.001***	26.21 ± 3.56	22.99 ± 2.78	<0.001***
BF%	26.79 ± 3.58	26.76 ± 4.52	0.953	36.56 ± 4.47	36.43 ± 4.46	0.830
WHR	1.25 ± 0.19	1.15 ± 0.18	<0.001***	1.08 ± 0.15	1.03 ± 0.16	0.008*
FPG (mmol/L)	7.59 ± 2.54	7.21 ± 2.45	0.224	7.46 ± 2.53	7.36 ± 3.21	0.775
TC (mmol/L)	4.00 ± 1.07	3.85 ± 1.05	0.253	4.45 ± 1.17	4.44 ± 1.14	0.973
TG (mmol/L)	1.75 ± 1.43	1.40 ± 1.22	0.035*	1.67 ± 1.04	1.40 ± 0.90	0.034*
HDL-C (mmol/L)	1.06 ± 0.27	1.09 ± 0.26	0.358	1.20 ± 0.29	1.31 ± 0.37	0.012*
LDL-C (mmol/L)	2.30 ± 0.80	2.26 ± 0.84	0.702	2.61 ± 0.91	2.57 ± 0.87	0.738
Serum albumin (g/L)	42.54 ± 3.63	41.62 ± 3.86	0.052	42.32 ± 3.07	40.84 ± 3.84	<0.001***
Serum creatinine (µmol/L)	80.97 ± 20.98	75.06 ± 22.02	0.029*	63.96 ± 16.07	60.31 ± 19.09	0.104
HbA1c	8.42 ± 1.73	8.83 ± 1.96	0.082	8.53 ± 1.67	8.89 ± 1.90	0.115
FCP (ng/mL)	1.98 ± 2.09	1.39 ± 1.00	0.006**	1.58 ± 0.89	1.50 ± 0.93	0.476
FT3 (pmol/L)	4.77 ± 0.54	4.70 ± 0.67	0.385	4.50 ± 0.60	4.40 ± 0.56	0.181
FT4 (pmol/L)	16.63 ± 2.62	16.79 ± 2.74	0.631	16.17 ± 2.90	16.12 ± 2.63	0.902
TSH (μIU/mL)	1.86 ± 1.00	1.78 ± 1.21	0.582	3.04 ± 6.63	2.27 ± 1.85	0.271
Serum calcium (mmol/L)	2.28 ± 0.09	2.26 ± 0.09	0.104	2.31 ± 0.09	2.27 ± 0.11	<0.001***
iPTH (pg/mL)	37.39 ± 15.14	32.67 ± 12.51	0.008**	38.53 ± 14.66	37.20 ± 14.09	0.477
DSPN (n, %)	55(50.00%)	111(70.70%)	<0.001***	77(48.73%)	74(77.08%)	<0.001***
DR (n, %)	58(52.73%)	87(55.41%)	0.664	104(65.82%)	56(58.33%)	0.231
DKD (n, %)	11(10.00%)	9(5.73%)	0.192	14(8.86%)	9(9.38%)	0.890
Osteoporosis (n, %)	11(10.00%)	20(12.74%)	0.492	44(27.85%)	43(44.79%)	0.006**
Antidiabetic medication use						
Insulin (n, %)	68(61.82%)	53(33.76%)	<0.001***	99(62.66%)	47(48.96%)	0.032*
Sulfonylureas (n, %)	34(30.91%)	71(45.22%)	0.018*	43(27.22%)	39(40.63%)	0.027*
DPP-4 inhibitors (n, %)	39(35.45%)	37(23.57%)	0.034*	53(33.54%)	19(19.79%)	0.018*

*represents *P* < 0.05, **represents *P* < 0.01, ***represents *P* < 0.001.

### 3.2 Associations of reduced skeletal muscle mass with clinical characteristics

**Figure 1:**
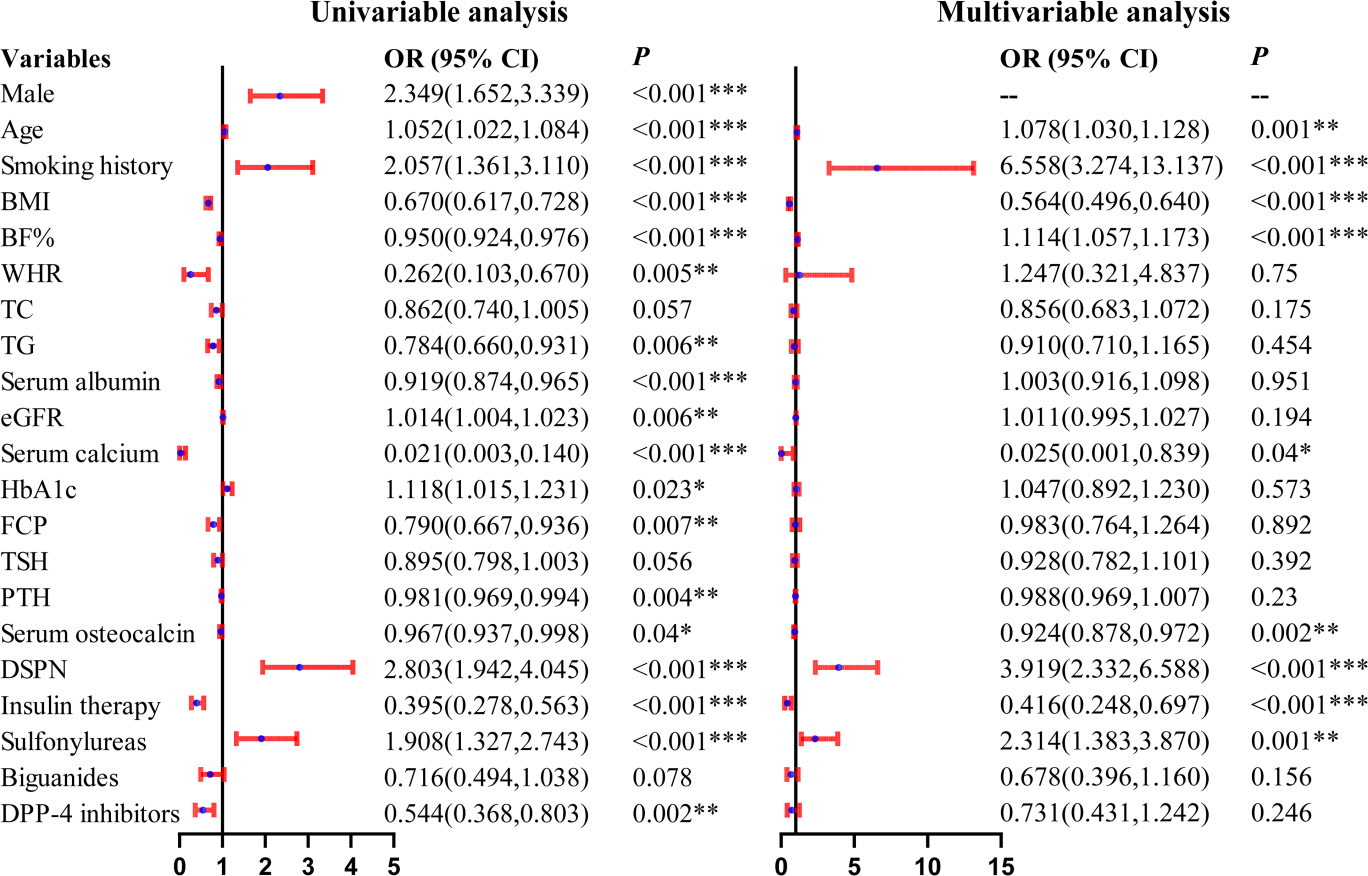
Forest Plot of Univariable and Multivariable Logistic Regression Analyses for Reduced Muscle Mass in Elderly Patients With T2DM (without sex stratification). *represents *P* < 0.05, **represents *P* < 0.01, ***represents *P* < 0.001.

In the overall study population, univariable logistic regression analysis identified several factors associated with reduced skeletal muscle mass, including male sex, older age, smoking history, lower BMI, BF%, and WHR, as well as lower serum albumin and calcium levels. After multivariable adjustment, older age (OR = 1.078, 95% CI: 1.030-1.128, *P* = 0.001), smoking history (OR = 6.558, 95% CI: 3.274-13.137, *P* < 0.001), higher BF% (OR = 1.114, 95% CI: 1.057-1.173, *P* < 0.001), diabetic neuropathy (OR = 3.919, 95% CI: 2.332-6.588, *P* < 0.001), and sulfonylurea use (OR = 2.314, 95% CI: 1.383-3.870, *P* = 0.001) were independently associated with an increased risk of reduced skeletal muscle mass. In contrast, higher BMI (OR = 0.564, 95% CI: 0.496-0.640, *P* < 0.001), higher serum calcium (OR = 0.025, 95% CI: 0.001-0.839, *P* = 0.040) and osteocalcin levels (OR = 0.924, 95% CI: 0.878-0.972, *P* = 0.002), as well as insulin use (OR = 0.416, 95% CI: 0.248-0.697, *P* < 0.001), were independently associated with a decreased risk. Given the sex-specific diagnostic criteria for low muscle mass and the strong collinearity between sex and multiple body composition-related variables, sex was therefore excluded from the final multivariable regression model. The corresponding results are presented in **Figure 1**.

**Figure 2:**
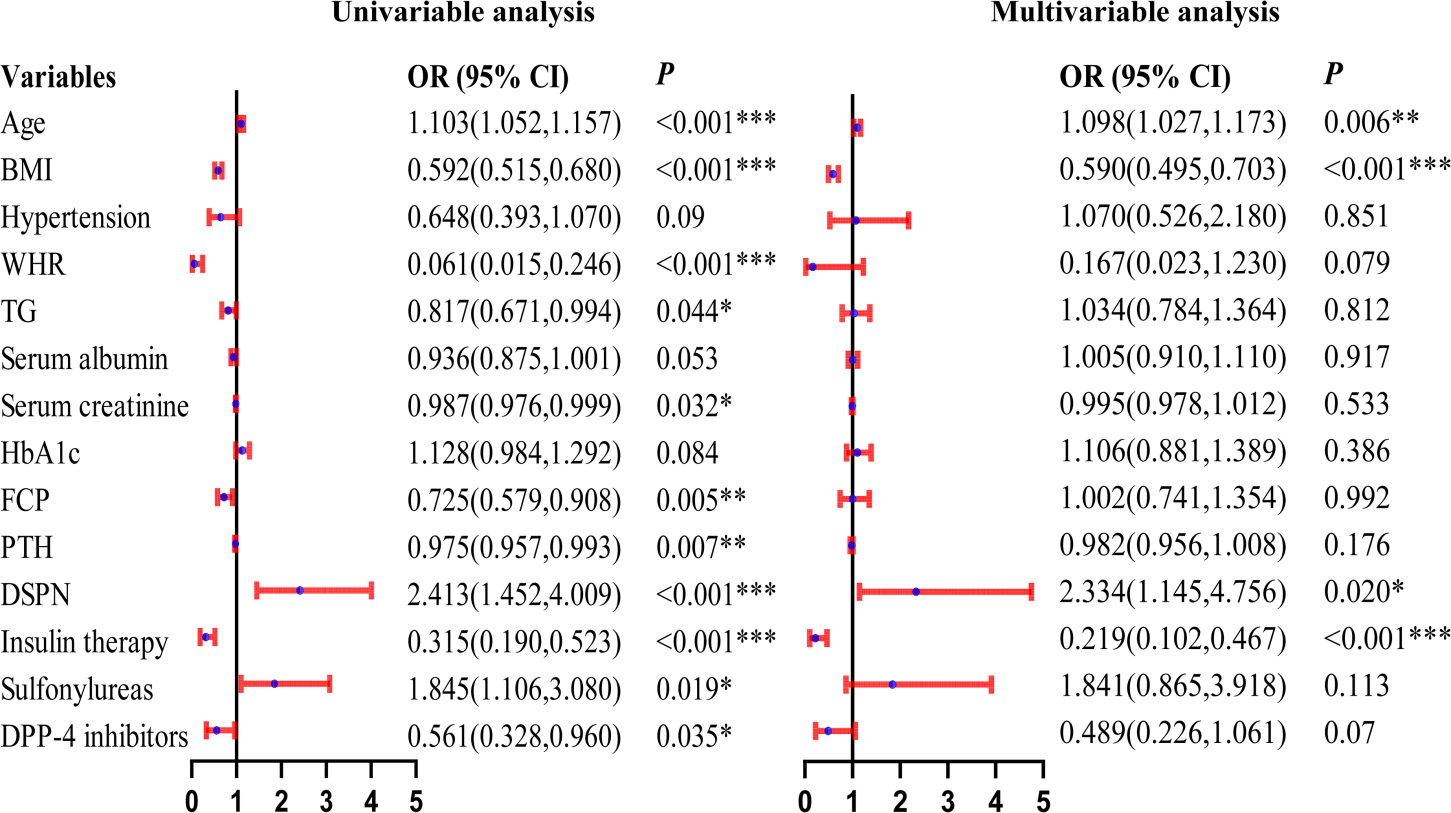
Forest Plot of Univariable and Multivariable Logistic Regression Analyses for Reduced Muscle Mass in Elderly Male Patients With T2DM. *represents *P* < 0.05, **represents *P* < 0.01, ***represents *P* < 0.001.

**Figure 3:**
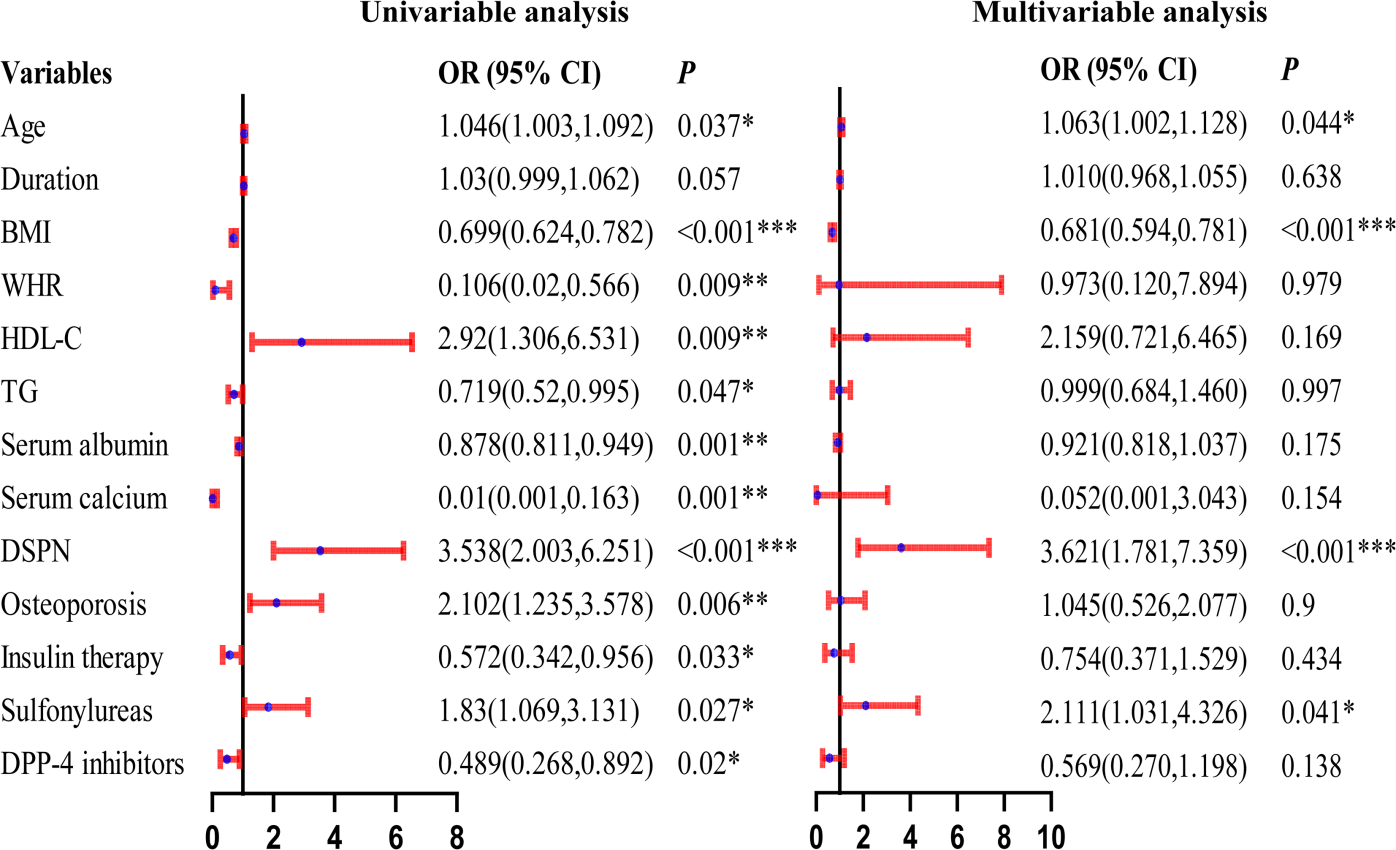
Forest Plot of Univariable and Multivariable Logistic Regression Analyses for Reduced Muscle Mass in Elderly Female Patients With T2DM. *represents *P* < 0.05, **represents *P* < 0.01, ***represents *P* < 0.001.

After sex stratification, distinct patterns of independent associations with reduced skeletal muscle mass were observed. In men, older age (OR = 1.098, 95% CI: 1.027-1.173, *P* = 0.006) and diabetic neuropathy (OR = 2.334, 95% CI: 1.145-4.756, *P* = 0.020) were independently associated with an increased risk, whereas higher BMI (OR = 0.590, 95% CI: 0.495-0.703, *P* < 0.001) and insulin use (OR = 0.219, 95% CI: 0.102-0.467, *P* < 0.001) were associated with a lower risk. In women, older age (OR = 1.063, 95% CI: 1.002-1.128, *P* = 0.044), diabetic neuropathy (OR = 3.621, 95% CI: 1.781-7.359, *P* < 0.001), and sulfonylurea use (OR = 2.111, 95% CI: 1.031-4.326, *P* = 0.041) were independently associated with an increased risk, while higher BMI (OR = 0.681, 95% CI: 0.594-0.781, *P* < 0.001) remained protective. These results are shown in **Figures 2**, **3**.

### 3.3 Predictive performance of relevant factors: ROC curve analysis

**Figure 4:**
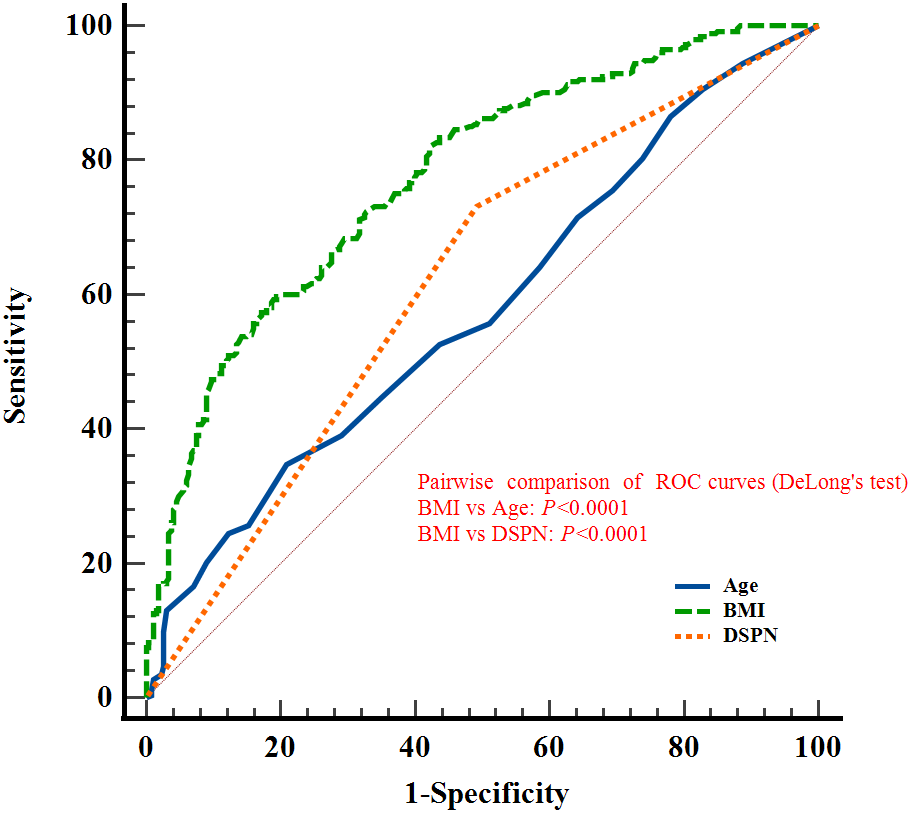
ROC curves of Age, BMI, and DSPN for discriminating reduced skeletal muscle mass in elderly patients with T2DM (without sex stratification).

In the overall population, ROC curve analyses were performed for age, BMI, and DSPN, which were consistently identified as independent predictors of reduced skeletal muscle mass in both overall and sex-stratified multivariable models. The AUCs were 0.577 (95% CI: 0.533-0.620) for age, 0.775 (0.736-0.810) for BMI, and 0.619 (0.576-0.661) for DSPN. The corresponding sensitivities were 34.78%, 59.68%, and 73.12%, with specificities of 79.10%, 80.60%, and 50.75%, respectively (all *P* < 0.05; **Figure 4**; **Table 3**). The *P* values indicate whether the AUCs are significantly greater than 0.5.

**Table 3:** ROC analysis of risk factors for discriminating reduced muscle mass.

General population	AUC (95%CI)	Sensitivity	Specificity	Youden index	*P* value
Age	0.577(0.533,0.620)	34.78%	79.10%	0.1389	0.002**
BMI	0.775(0.736,0.810)	59.68%	80.60%	0.4028	<0.0001****
DSPN	0.619(0.576,0.661)	73.12%	50.75%	0.2387	<0.0001****
Male					
Age	0.636(0.576,0.694)	35.03%	85.45%	0.2049	0.0001***
BMI	0.815(0.763,0.859)	84.71%	65.45%	0.5017	<0.0001****
DSPN	0.604(0.542,0.663)	70.70%	50.00%	0.2070	0.0006***
Insulin therapy	0.640(0.580,0.698)	66.24%	61.82%	0.2806	<0.0001****
Female					
Age	0.570(0.507,0.632)	43.75%	72.78%	0.1653	0.0634
BMI	0.763(0.705,0.814)	64.58%	77.85%	0.4243	<0.0001****
DSPN	0.642(0.579,0.701)	77.08%	51.27%	0.2835	<0.0001****
Sulfonylureas	0.567(0.504,0.629)	40.63%	72.78%	0.1341	0.0296*

The *P* value indicates whether the AUC is significantly greater than 0.5.

*represents *P* < 0.05, **represents *P* < 0.01, ***represents *P* < 0.001, ****represents *P* < 0.0001.

**Figure 5:**
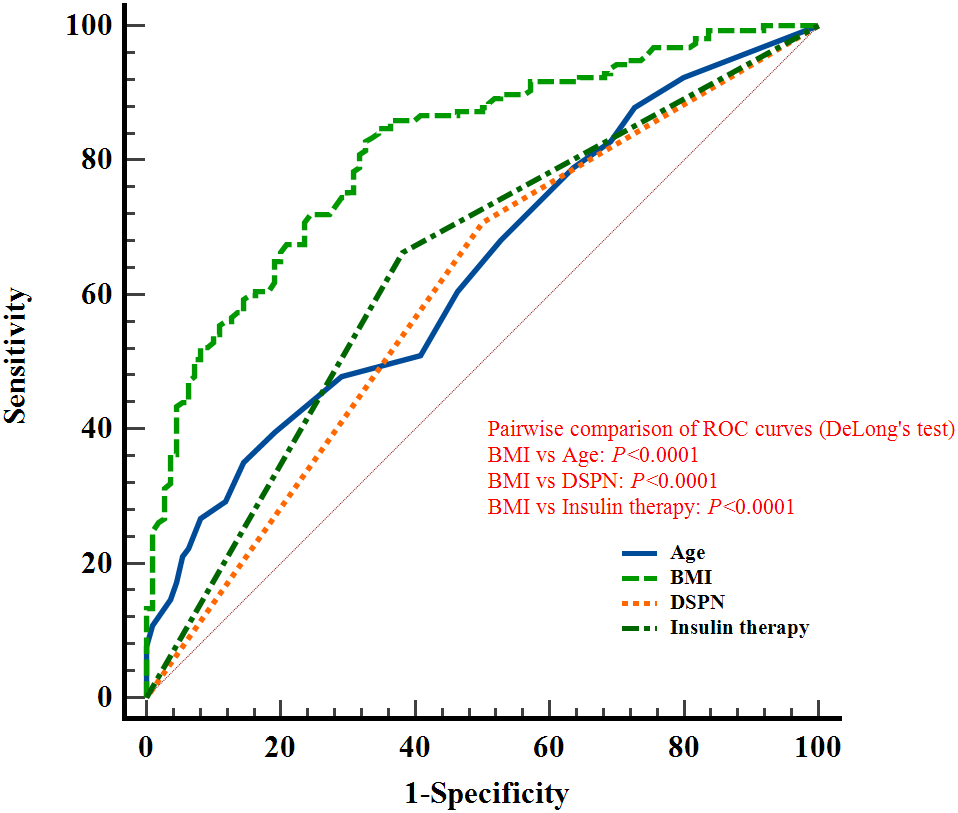
ROC curves of Age, BMI, DSPN, and Insulin therapy for discriminating reduced skeletal muscle mass in elderly male patients with T2DM.

In male patients, ROC analyses for age, BMI, DSPN, and insulin therapy yielded AUCs of 0.636 (95% CI: 0.576-0.694), 0.815 (0.763-0.859), 0.604 (0.542-0.663), and 0.640 (0.580-0.698), respectively, for discriminating low muscle mass. The corresponding sensitivities were 35.03%, 84.71%, 70.70%, and 66.24%, with specificities of 85.45%, 65.45%, 50.00%, and 61.82% (all *P* < 0.05; **Figure 5**; **Table 3**).

**Figure 6:**
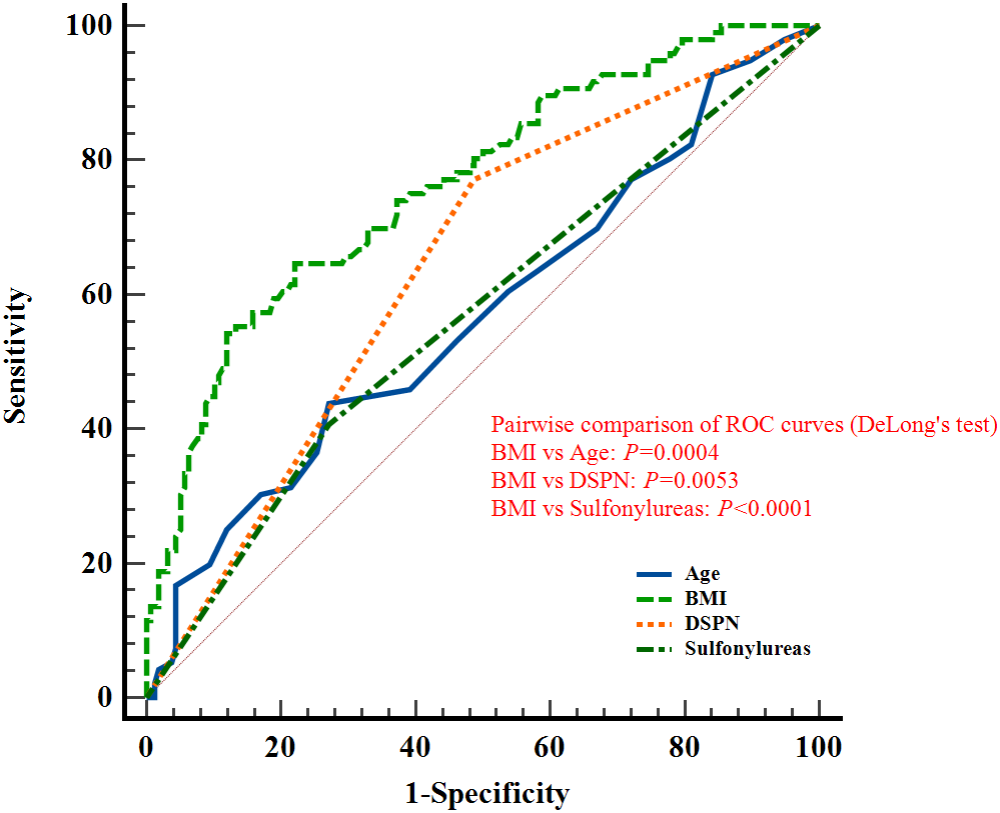
ROC curves of Age, BMI, DSPN, and Sulfonylureas for discriminating reduced skeletal muscle mass in elderly female patients with T2DM.

In female patients, ROC analyses yielded AUCs of 0.570 (95% CI: 0.507-0.632) for age, 0.763 (0.705-0.814) for BMI, 0.642 (0.579-0.701) for DSPN, and 0.567 (0.504-0.629) for sulfonylurea use. The corresponding sensitivities were 43.75%, 64.58%, 77.08%, and 40.63%, with specificities of 72.78%, 77.85%, 51.27%, and 72.78%. BMI, DSPN, and sulfonylurea use showed statistically significant discriminatory ability (*P* < 0.05; **Figure 6**; **Table 3**).

Pairwise comparisons using DeLong’s test confirmed that the AUC of BMI was significantly greater than those of the other factors in the overall population as well as in male and female subgroups. (*P* < 0.05; **Figures 4**-**6**). The *P* values indicate whether the differences in AUCs between BMI and other individual factors were statistically significant.

## 4 Discussion

In this retrospective cohort of 521 elderly patients with T2DM, age, BMI, and DSPN emerged as independent determinants of reduced skeletal muscle mass, underscoring the multifactorial nature of muscle loss in this population.

Age and BMI showed significant associations with low muscle mass, with higher BMI exerting a protective effect, in line with previous findings (16–18). In our cohort, a higher BMI was associated with a lower risk of muscle mass loss and demonstrated superior discriminative performance across subgroups, supporting its potential utility as a simple clinical indicator for identifying individuals at risk of low muscle mass. Although BMI is traditionally regarded as a surrogate for adiposity, in elderly patients with T2DM, insulin resistance, chronic low-grade inflammation, and impaired anabolic signaling may collectively promote preferential loss of lean mass. Consequently, a lower BMI in this population is more likely to reflect skeletal muscle loss rather than fat loss and may serve as a clinical marker of frailty and reduced muscle reserves (19–21). Compared with BMI, BF% more accurately reflects overall adiposity. Excess adiposity may promote skeletal muscle loss and functional impairment through oxidative stress, chronic low-grade inflammation, and insulin resistance–related metabolic dysregulation (22, 23). Consistent with this framework, BF% was independently associated with low muscle mass in the overall population; however, this association was attenuated and did not reach statistical significance after sex stratification, potentially due to sex-related differences in body composition and limited statistical power in subgroup analyses. Characterized by concurrent loss of lean mass and increased adiposity, sarcopenic obesity represents a distinct clinical phenotype that warrants further investigation, with reported prevalence ranging from 10% to 23% (24–26).

In this study, neither FPG nor HbA1c was significantly associated with skeletal muscle mass, suggesting that glycemic control alone may be insufficient to account for the mechanisms underlying muscle preservation in elderly patients with T2DM. In contrast, FCP levels were significantly lower in patients—particularly men—with reduced muscle mass, implicating impaired endogenous insulin secretion in muscle loss. As a key anabolic hormone, insulin promotes skeletal muscle protein synthesis via phosphatidylinositol 3-kinase (PI3K) pathway activation, whereas reduced insulin availability or insulin resistance may impair protein turnover and promote muscle atrophy (27, 28). Notably, this association was not evident in women, further supporting sex-specific differences in muscle mass regulation (29).

Diabetic neuropathy, affecting approximately 30–50% of individuals with T2DM, is increasingly recognized as a key contributor to accelerated skeletal muscle mass loss (30–32). Consistent with previous reports, our findings further demonstrated a significant association between diabetic neuropathy and reduced skeletal muscle mass, supporting its potential utility as an early clinical indicator for identifying individuals at increased risk of muscle loss. Mechanistically, diabetic neuropathy involves progressive axonal and motor unit degeneration, reduced neurotrophin-3 expression, and impaired neuromuscular maintenance, which may collectively disrupt neuromuscular signaling and thereby compromise skeletal muscle glucose metabolism and protein synthesis (33–35). Collectively, these findings underscore the need for integrated management strategies targeting both metabolic control and peripheral nerve function to mitigate sarcopenia risk in this vulnerable population.

Bone metabolism and skeletal muscle health are closely linked through shared molecular pathways and coordinated endocrine regulation (36). In our cohort, changes in calcium–iPTH homeostasis and bone turnover markers appeared to be associated with reduced muscle mass, while higher serum calcium and osteocalcin levels showed independent protective associations against muscle loss. These findings support a contributory role of the bone–muscle axis in the development of sarcopenia among elderly patients with T2DM (37, 38). Notably, the observed sex-specific patterns suggest that hormonal modulation of bone–muscle crosstalk may underlie differential vulnerability, with estrogen deficiency in women potentially exacerbating bone resorption–related musculoskeletal signaling, while dysregulated calcium–iPTH balance in men may impair mineral homeostasis and contribute to muscle atrophy (39–41).

Recent studies suggest differential effects of antidiabetic medications on skeletal muscle health and body composition (42, 43). In our cohort, both insulin and sulfonylurea use were independently associated with reduced skeletal muscle mass in the overall population. Insulin, a key anabolic hormone, promotes protein synthesis and metabolic homeostasis (44). In contrast, sulfonylureas lower glucose levels by inhibiting adenosine triphosphate–sensitive potassium (KATP) channels, which are essential for skeletal muscle energy coupling; such inhibition may perturb cellular energy homeostasis and membrane potential, thereby potentially activating pathways implicated in muscle atrophy (45). Our findings further indicated sex-specific differences in susceptibility to low muscle mass, with male patients exhibiting greater vulnerability. Given the age-related decline in testosterone levels in men, attenuation of the testosterone–insulin-like growth factor-1 (IGF-1) axis may partly account for this association. Testosterone promotes IGF-1 synthesis, a key regulator of skeletal muscle and bone metabolism (46, 47), and age-related disruption of this hormonal axis may impair anabolic signaling and contribute to accelerated muscle loss in elderly men with T2DM.

This study has several limitations. First, the cross-sectional design precludes causal inference between low muscle mass and the associated risk factors. Second, as a single-center study, the findings may not be fully generalizable to the broader population of older adults with T2DM.

## 5 Conclusion

This study demonstrated that, among elderly patients with T2DM, older age, lower BMI, diabetic sensorimotor polyneuropathy (DSPN), and the use of insulin and sulfonylureas were independently associated with reduced skeletal muscle mass. Among these factors, BMI exhibited the greatest discriminative performance, highlighting its potential as a practical marker for identifying individuals at risk of muscle loss in this population.

## Data Availability

The data analyzed in this study is subject to the following licenses/restrictions: the datasets generated and analyzed during the current study are not publicly available due to patient privacy and institutional regulations but are available from the corresponding author upon reasonable request. Requests to access these datasets should be directed to Kaili Wang, wangkaili202201@163.com.
