# Supplementary material for: Determinants and predictive performance of reduced muscle mass in elderly patients with type 2 diabetes: a retrospective study
Source: Front Endocrinol (Lausanne). 2026 Mar 4;17:1746797. doi: 10.3389/fendo.2026.1746797 (PMC12995614; doi:10.3389/fendo.2026.1746797)
Supplement: Supplementary file 1 [file Table1.docx]

Table 1. Baseline Characteristics of Participants With and Without Low Muscle Mass

| Variables | Normal muscle mass | Low muscle Mass | *P* value |
| --- | --- | --- | --- |
| N | 268 | 253 |  |
| Male (n, %) | 110(41.04%) | 157(62.06%) | <0.001*** |
| Age (years) | 67.76±5.56 | 69.56±6.33 | <0.001*** |
| Duration of diabetes (years) | 15.88±7.76 | 16.96±8.54 | 0.128 |
| Hypertension (n, %) | 176(65.67%) | 149(58.89%) | 0.110 |
| CAD (n, %) | 119(44.40%) | 99(39.13%) | 0.223 |
| Smoking habit (n, %) | 47(17.54%) | 77(30.43%) | <0.001*** |
| Drinking habit (n, %) | 49(18.28%) | 51(20.16%) | 0.587 |
| BMI (kg/m^2^) | 26.42±3.24 | 23.36±2.54 | <0.001*** |
| BF% | 32.55±6.34 | 30.43±6.50 | <0.001*** |
| WHR | 1.15±0.19 | 1.10±0.18 | 0.005** |
| FPG (mmol/L) | 7.53±2.53 | 7.27±2.76 | 0.287 |
| TC (mmol/L) | 4.26±1.15 | 4.07±1.12 | 0.056 |
| TG (mmol/L) | 1.70±1.21 | 1.40±1.11 | 0.004** |
| HDL-C (mmol/L) | 1.14±0.29 | 1.18±0.32 | 0.222 |
| LDL-C (mmol/L) | 2.48±0.88 | 2.38±0.86 | 0.176 |
| Serum albumin (g/L) | 42.41±3.31 | 41.33±3.86 | <0.001*** |
| eGFR (mL/min/1.73m^2^) | 86.09±17.85 | 90.47±18.39 | 0.006** |
| Serum creatinine (µmol/L) | 70.94±20.05 | 69.47±22.11 | 0.424 |
| Serum BUN (mmol/L) | 6.29±1.94 | 6.30±2.09 | 0.954 |
| HbA1c | 8.49±1.69 | 8.85±1.94 | 0.022* |
| FCP (ng/mL) | 1.75±1.51 | 1.43±0.97 | 0.005** |
| FT3 (pmol/L) | 4.61±0.59 | 4.59±0.65 | 0.640 |
| FT4 (pmol/L) | 16.36±2.80 | 16.54±2.71 | 0.459 |
| TSH (μIU/mL) | 2.56±5.15 | 1.97±1.50 | 0.084 |
| iPTH (pg/mL) | 38.06±14.84 | 34.39±13.29 | 0.003** |
| Vitamin D (ng/mL) | 19.49±7.57 | 20.11±8.36 | 0.375 |
| Serum calcium (mmol/L) | 2.30±0.09 | 2.26±0.10 | <0.001*** |
| Osteocalcin (OC) (ng/mL) | 13.45±5.58 | 12.43±5.63 | 0.038* |
| β-CTX (ng/mL) | 0.38±0.18 | 0.39±0.21 | 0.427 |
| P1NP (ng/mL) | 45.20±18.24 | 43.92±21.57 | 0.464 |
| DSPN (n, %) | 132(49.25%) | 185(73.12%) | <0.001*** |
| DR (n, %) | 162(60.45%) | 143(56.52%) | 0.363 |
| DKD (n, %) | 25(9.33%) | 18(7.11%) | 0.359 |
| Osteoporosis (n, %) | 55(20.52%) | 63(23.51%) | 0.233 |
| Statins (n, %) | 152(56.72%) | 129(50.99%) | 0.190 |
| Antidiabetic medication use |  |  |  |
| Insulin (n, %) | 167(62.31%) | 100(39.53%) | <0.001*** |
| Sulfonylureas (n, %) | 77(28.73%) | 110(43.48%) | <0.001*** |
| Metformin (n, %) | 193(72.01%) | 164(64.82%) | 0.070 |
| α-glucosidase inhibitors (n, %) | 92(34.33%) | 83(32.81%) | 0.713 |
| SGLT2 inhibitors (n, %) | 74(27.61%) | 70(27.67%) | 0.989 |
| DPP-4 inhibitors (n, %) | 92(34.33%) | 56(22.13%) | 0.002** |
| GLP-1 receptor agonists (n, %) | 13(4.85%) | 11(4.35%) | 0.784 |

CAD, coronary artery disease; BMI, body mass index; BF%, body fat percentage; WHR, waist-to-hip ratio; FPG, fasting plasma glucose; TC, total cholesterol; TG, triglycerides; HDL-C, high-density lipoprotein cholesterol; LDL-C, low-density lipoprotein cholesterol; eGFR, estimated glomerular filtration rate; BUN, blood urea nitrogen; HbA1c, glycated hemoglobin; FCP, fasting C-peptide; FT3, free triiodothyronine; FT4, free thyroxine; TSH, thyroid-stimulating hormone; iPTH, intact parathyroid hormone; β-CTX, β-C-terminal telopeptide of type I collagen; P1NP, N-terminal propeptide of type I procollagen; DSPN, diabetic sensorimotor polyneuropathy; DR, diabetic retinopathy; DKD, diabetic kidney disease; SGLT2, sodium-glucose cotransporter 2; DPP-4, dipeptidyl peptidase-4; GLP-1: glucagon-like peptide-1

*represents *P* <0.05, **represents *P* <0.01, ***represents *P* <0.001.

Table 2. Baseline Characteristics of Participants Stratified by Sex and Skeletal Muscle Mass

| Variables | Male(n=267) | | | Female(n=254) | | |
| --- | --- | --- | --- | --- | --- | --- |
|  | Normal muscle mass | Low muscle Mass | *P* value | Normal muscle mass | Low muscle Mass | *P* value |
|  |  |  |  |  |  |  |
| N | 110 | 157 | -- | 158 | 96 | -- |
| Age (years) | 65.86±4.74 | 68.85±6.17 | <0.001*** | 69.08±5.71 | 70.72±6.44 | 0.035* |
| Duration of diabetes (years) | 16.19±7.68 | 16.49±8.10 | 0.762 | 15.66±7.83 | 17.75±9.21 | 0.066 |
| Hypertension (n, %) | 71(64.55%) | 85(54.14%) | 0.090 | 105(66.46%) | 64(66.67%) | 0.972 |
| CAD (n, %) | 47(42.73%) | 61(38.85%) | 0.526 | 72(45.57%) | 38(39.58%) | 0.351 |
| Smoking habit (n, %) | 46(41.82%) | 75(47.77%) | 0.336 | 1(0.63%) | 2(2.08%) | 0.559 |
| Drinking habit (n, %) | 46(41.82%) | 51(32.48%) | 0.119 | 3(1.90%) | 0(0%) | 0.292 |
| BMI (kg/m^2^) | 26.71±2.69 | 23.58±2.37 | <0.001*** | 26.21±3.56 | 22.99±2.78 | <0.001*** |
| BF% | 26.79±3.58 | 26.76±4.52 | 0.953 | 36.56±4.47 | 36.43±4.46 | 0.830 |
| WHR | 1.25±0.19 | 1.15±0.18 | <0.001*** | 1.08±0.15 | 1.03±0.16 | 0.008** |
| FPG (mmol/L) | 7.59±2.54 | 7.21±2.45 | 0.224 | 7.46±2.53 | 7.36±3.21 | 0.775 |
| TC (mmol/L) | 4.00±1.07 | 3.85±1.05 | 0.253 | 4.45±1.17 | 4.44±1.14 | 0.973 |
| TG (mmol/L) | 1.75±1.43 | 1.40±1.22 | 0.035* | 1.67±1.04 | 1.40±0.90 | 0.034* |
| HDL-C (mmol/L) | 1.06±0.27 | 1.09±0.26 | 0.358 | 1.20±0.29 | 1.31±0.37 | 0.012* |
| LDL-C (mmol/L) | 2.30±0.80 | 2.26±0.84 | 0.702 | 2.61±0.91 | 2.57±0.87 | 0.738 |
| Serum albumin (g/L) | 42.54±3.63 | 41.62±3.86 | 0.052 | 42.32±3.07 | 40.84±3.84 | <0.001*** |
| eGFR (mL/min/1.73m^2^) | 89.81±20.48 | 93.02±18.97 | 0.189 | 83.50±15.30 | 86.32±16.68 | 0.170 |
| Serum creatinine (µmol/L) | 80.97±20.98 | 75.06±22.02 | 0.029* | 63.96±16.07 | 60.31±19.09 | 0.104 |
| Serum BUN (mmol/L) | 6.81±2.03 | 6.56±2.09 | 0.328 | 5.93±1.80 | 5.88±2.03 | 0.834 |
| HbA1c | 8.42±1.73 | 8.83±1.96 | 0.082 | 8.53±1.67 | 8.89±1.90 | 0.115 |
| FCP (ng/mL) | 1.98±2.09 | 1.39±1.00 | 0.006** | 1.58±0.89 | 1.50±0.93 | 0.476 |
| FT3 (pmol/L) | 4.77±0.54 | 4.70±0.67 | 0.385 | 4.50±0.60 | 4.40±0.56 | 0.181 |
| FT4 (pmol/L) | 16.63±2.62 | 16.79±2.74 | 0.631 | 16.17±2.90 | 16.12±2.63 | 0.902 |
| TSH (μIU/mL) | 1.86±1.00 | 1.78±1.21 | 0.582 | 3.04±6.63 | 2.27±1.85 | 0.271 |
| iPTH (pg/mL) | 37.39±15.14 | 32.67±12.51 | 0.008** | 38.53±14.66 | 37.20±14.09 | 0.477 |
| Vitamin D (ng/mL) | 21.72±7.79 | 21.22±8.72 | 0.631 | 17.94±7.03 | 18.29±7.43 | 0.704 |
| Serum calcium (mmol/L) | 2.28±0.09 | 2.26±0.09 | 0.104 | 2.31±0.09 | 2.27±0.11 | <0.001*** |
| Osteocalcin (OC) (ng/mL) | 11.80±4.61 | 11.48±4.99 | 0.595 | 14.60±5.92 | 13.98±6.26 | 0.428 |
| β-CTX (ng/mL) | 0.34±0.17 | 0.36±0.20 | 0.358 | 0.40±0.18 | 0.44±0.22 | 0.126 |
| P1NP (ng/mL) | 40.44±15.08 | 41.77±20.90 | 0.567 | 48.52±19.51 | 47.43±22.29 | 0.684 |
| DSPN (n, %) | 55(50.00%) | 111(70.70%) | <0.001*** | 77(48.73%) | 74(77.08%) | <0.001*** |
| DR (n, %) | 58(52.73%) | 87(55.41%) | 0.664 | 104(65.82%) | 56(58.33%) | 0.231 |
| DKD (n, %) | 11(10.00%) | 9(5.73%) | 0.192 | 14(8.86%) | 9(9.38%) | 0.890 |
| Osteoporosis (n, %) | 11(10.00%) | 20(12.74%) | 0.492 | 44(27.85%) | 43(44.79%) | 0.006** |
| Statins (n, %) | 62(56.36%) | 81(51.59%) | 0.442 | 90(56.96%) | 48(50.00%) | 0.280 |
| Antidiabetic medication use |  |  |  |  |  |  |
| Insulin (n, %) | 68(61.82%) | 53(33.76%) | <0.001*** | 99(62.66%) | 47(48.96%) | 0.032* |
| Sulfonylureas (n, %) | 34(30.91%) | 71(45.22%) | 0.018* | 43(27.22%) | 39(40.63%) | 0.027* |
| Metformin (n, %) | 80(72.73%) | 103(65.61%) | 0.217 | 113(71.52%) | 61(63.54%) | 0.184 |
| α-glucosidase inhibitors (n, %) | 40(36.36%) | 46(29.30%) | 0.224 | 52(32.91%) | 37(38.54%) | 0.362 |
| SGLT2 inhibitors (n, %) | 32(29.09%) | 49(31.21%） | 0.711 | 42(26.58%) | 21(21.88%) | 0.400 |
| DPP-4 inhibitors (n, %) | 39(35.45%) | 37(23.57%) | 0.034* | 53(33.54%) | 19(19.79%) | 0.018* |
| GLP-1 receptor agonists (n, %) | 9(8.18%) | 7(4.46%) | 0.207 | 4(2.53%) | 4(4.17%) | 0.481 |

*represents *P* <0.05, **represents *P* <0.01, ***represents *P* <0.001.
